# Comparison of mHealth and Face-to-Face Interventions for Smoking Cessation Among People Living With HIV: Meta-Analysis

**DOI:** 10.2196/mhealth.9329

**Published:** 2019-01-07

**Authors:** Olalekan A Uthman, Chidozie U Nduka, Mustapha Abba, Rocio Enriquez, Helena Nordenstedt, Fred Nalugoda, Andre P Kengne, Anna M Ekström

**Affiliations:** 1 Warwick-Centre for Applied Health Research and Delivery Division of Health Sciences University Warwick Coventry United Kingdom; 2 Division of Health Sciences University of Warwick Coventry United Kingdom; 3 Global and Sexual Health Department of Public Health Karolinska Institutet Stockholm United Kingdom; 4 Uganda Virus Research Institute Rakai Health Sciences Program Rakai Uganda; 5 Non-Communicable Diseases Research Unit South African Medical Research Council Cape Town South Africa

**Keywords:** HIV, mHealth, smoking cessation

## Abstract

**Background:**

The prevalence of smoking among people living with HIV (PLHIV) is higher than that reported in the general population, and it is a significant risk factor for noncommunicable diseases in this group. Mobile phone interventions to promote healthier behaviors (mobile health, mHealth) have the potential to reach a large number of people at a low cost. It has been hypothesized that mHealth interventions may not be as effective as face-to-face strategies in achieving smoking cessation, but there is no systematic evidence to support this, especially among PLHIV.

**Objective:**

This study aimed to compare two modes of intervention delivery (mHealth vs face-to-face) for smoking cessation among PLHIV.

**Methods:**

Literature on randomized controlled trials (RCTs) investigating effects of mHealth or face-to-face intervention strategies on short-term (4 weeks to <6 months) and long-term (≥6 months) smoking abstinence among PLHIV was sought. We systematically reviewed relevant RCTs and conducted pairwise meta-analyses to estimate relative treatment effects of mHealth and face-to-face interventions using standard care as comparison. Given the absence of head-to-head trials comparing mHealth with face-to-face interventions, we performed adjusted indirect comparison meta-analyses to compare these interventions.

**Results:**

A total of 10 studies involving 1772 PLHIV met the inclusion criteria. The average age of the study population was 45 years, and women comprised about 37%. In the short term, mHealth-delivered interventions were significantly more efficacious in increasing smoking cessation than no intervention control (risk ratio, RR, 2.81, 95% CI 1.44-5.49; n=726) and face-to-face interventions (RR 2.31, 95% CI 1.13-4.72; n=726). In the short term, face-to-face interventions were no more effective than no intervention in increasing smoking cessation (RR 1.22, 95% CI 0.94-1.58; n=1144). In terms of achieving long-term results among PLHIV, there was no significant difference in the rates of smoking cessation between those who received mHealth-delivered interventions, face-to-face interventions, or no intervention. Trial sequential analysis showed that only 15.16% (726/1304) and 5.56% (632/11,364) of the required information sizes were accrued to accept or reject a 25% relative risk reduction for short- and long-term smoking cessation treatment effects. In addition, sequential monitoring boundaries were not crossed, indicating that the cumulative evidence may be unreliable and inconclusive.

**Conclusions:**

Compared with face-to-face interventions, mHealth-delivered interventions can better increase smoking cessation rate in the short term. The evidence that mHealth increases smoking cessation rate in the short term is encouraging but not sufficient to allow a definitive conclusion presently. Future research should focus on strategies for sustaining smoking cessation treatment effects among PLHIV in the long term.

## Introduction

The introduction of effective antiretroviral therapy has resulted in a marked reduction in AIDS-related mortality worldwide. Patterns of morbidity and mortality have shifted from AIDS-related opportunistic infections to age-related comorbidities; moreover, it is now recognized that people living with HIV (PLHIV) are at increased risk of developing cardiovascular disease [1,2]. This increased risk is likely to be of multifactorial origin [3]: the disease and its treatment. Moreover, PLHIV are predisposed to engage in unhealthy behaviors [1,2].

Tobacco use is the single most common cause of preventable death worldwide and an important modifiable risk factor for several chronic conditions, including coronary heart diseases, chronic obstructive pulmonary diseases, and certain cancers [4]. About 15% of the world’s population smoked tobacco in 2015 [5]. However, prevalence estimates of tobacco use in vulnerable populations are much higher: 32% among people with mental health disorders [6], 73% among homeless people [7], 77% among substance abusers [8], and 84% among prisoners [9]. Of note, the prevalence of smoking in PLHIV ranges between 50% and 70% [10], and like other vulnerable groups, success rates of quitting attempts and sustained abstinence are much lower than in the general population [11]. Smoking for stress relief, inadequate support from health service providers, and high smoking acceptance rates among communities of PLHIV are among the perceived barriers to abstinence in this high-risk group, and these considerably differ from self-reported barriers in apparently healthy populations without known chronic conditions [11]. For these reasons, intervention strategies for smoking cessation in the general population may not be as effective in HIV-positive populations. Although there have been several reports on the effectiveness of smoking cessation interventions among PLHIV [12], there are still questions left unanswered, notably about the mode of intervention delivery and its impact on smoking cessation. Short message service (SMS) text messages and other phone-based strategies have the potential to be more cost effective in service delivery than face-to-face contact, but it has been hypothesized that such mobile health (mHealth) strategies might be less effective or no different in terms of achieving smoking abstinence [12]. mHealth services provide unique opportunities for delivering smoking cessation interventions to large number of people at a low cost. However, there is no systematic evidence to substantiate this hypothesis. Therefore, we first sought to review all existing literature investigating mHealth and face-to-face interventions for smoking cessation among PLHIV. Second, we examined whether the required amount of information has been reached to confidently conclude that mHealth is more effective than no mHealth and that future trials need not examine this question any longer.

## Methods

### Information Sources and Search Strategy

We conducted searches on the following major databases: Embase, Medical Literature Analysis and Retrieval System Online, the Cochrane Library Central Register of Controlled Trials, the ClinicalTrials.gov registry, and cross-references of relevant articles for randomized controlled trials (RCTs) investigating the effectiveness of smoking cessation interventions among HIV-positive smokers and published until up to May 2018. Multimedia Appendix 1 shows the database search strategy, including the search term combinations used.

### Selection Criteria

We evaluated each identified study against the following predetermined selection criteria: *Types of population* (PLHIV); *types of intervention and comparator* (face-to-face counseling, mHealth-delivered intervention, or no intervention country group); *types of outcome* (smoking abstinence); and *study design* (RCTs).

### Selection of Studies

Two authors (CUN and MA) screened the titles and abstracts of all the potential studies we identified as a result of the search and coded them as “retrieve” (eligible or potentially eligible or unclear) or as “do not retrieve.” Any disagreements were arbitrated by a third author (OAU). Subsequently, CUN and MA assessed the full-text study reports to confirm their eligibility for inclusion while noting the reasons for excluding studies considered ineligible for the meta-analysis. Again, any disagreements were resolved following discussions with OAU.

### Data Extraction

CUN and MA extracted demographic and clinical data from the included studies where available. Data on trial design, sample size, mean age, proportion of women, average daily number of cigarettes, interventions, outcomes, and follow-up durations were extracted. Any disagreements were resolved following discussions with OAU.

### Outcome Measures

The main outcome was short-term smoking abstinence, which has been defined as abstinence of at least 4 weeks duration, but less than 6 months after the intervention was initiated [12]. The secondary outcome was smoking abstinence of at least 6 months duration (long-term abstinence) [13].

### Risk of Bias Assessment

CUN and MA judged the risk of bias in each included study using the Cochrane risk of bias assessment tool, which includes the following domains: randomization sequence generation (selection bias); allocation concealment (selection bias); blinding of participants, providers, (performance bias) and outcome assessors (detection bias); completeness of outcome data (attrition bias); and selective outcome reporting (reporting bias). Each RCT was classified as having “high,” “low,” or “unclear” risk of bias in each domain [14]. OAU resolved any differences in the assessments.

### Statistical Analysis

**Figure 1:**
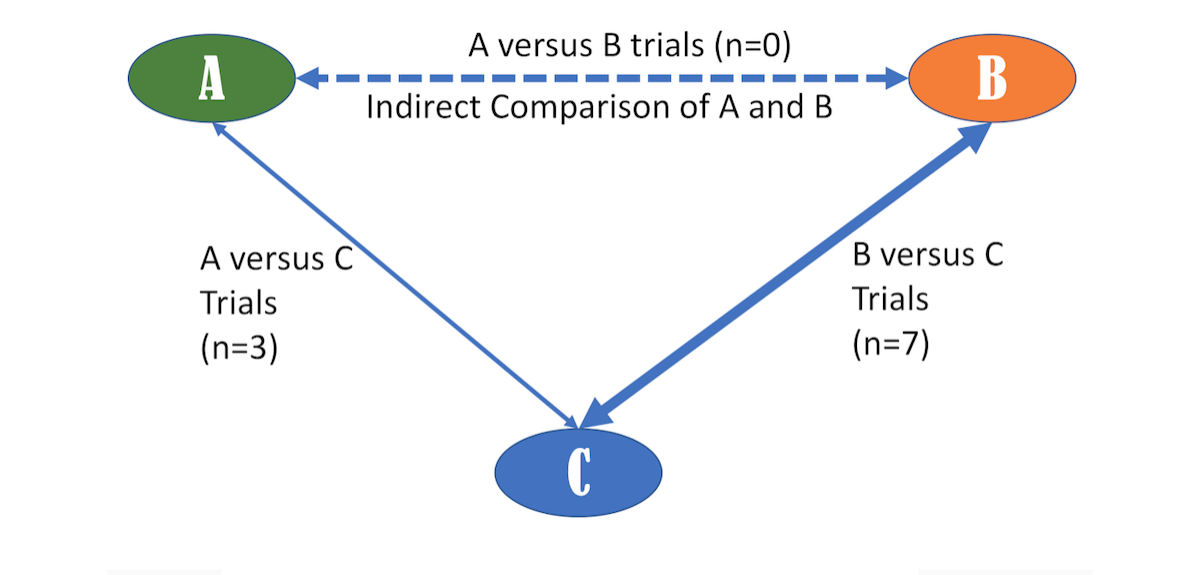
Adjusted indirect comparison network meta-analysis framework. A: mhealth delivered; B: face to face; C: standard of care.

We adopted an adjusted indirect comparison meta-analysis [15-18], a logical extension of standard meta-analysis to infer relative effectiveness of mHealth-delivered versus face-to-face interventions when the direct “head-to-head” evidence is lacking, that is, not directly addressed within any of the included trials. To illustrate this, in a situation where we have 3 treatments A (mHealth), B (face-to-face), and C (no intervention control), A and C have been compared in RCTs; B and C have been compared in other RCTs; and A and B had not been directly compared. The approach enabled the indirect comparisons (eg, A vs B) constructed from 2 trials that have one treatment in common to be incorporated (eg, A vs C and B vs C; Figure 1). Using Bucher adjusted indirect comparison method, the treatment effect for *T*_*AB*_ can be calculated by using the following equation:


*T_AB_=T_AC_–T_BC_*

where *T* represents the treatment effect (eg, log risk ratio, RR) between the 2 interventions. SE is calculated as follows:


*SE(T_AB_)=√(SE(T_AC_)^2^–SE(T_BC_)^2^)*

All data were analyzed using R package “stats” (version 3.2.2). As part of the primary analysis, subgroup analysis was conducted based on the intensity and duration of follow-up period. Analysis was performed separately for short-term (4 weeks to <6 months) and long-term (≥6 months) smoking abstinence. We also quantified heterogeneity by computing the *I*^2^ statistic; a value greater than 50% implied that the treatment estimates were considerably heterogeneous across the included studies. The pooled treatment estimates were reported using RRs and 95% CIs.

**Figure 2:**
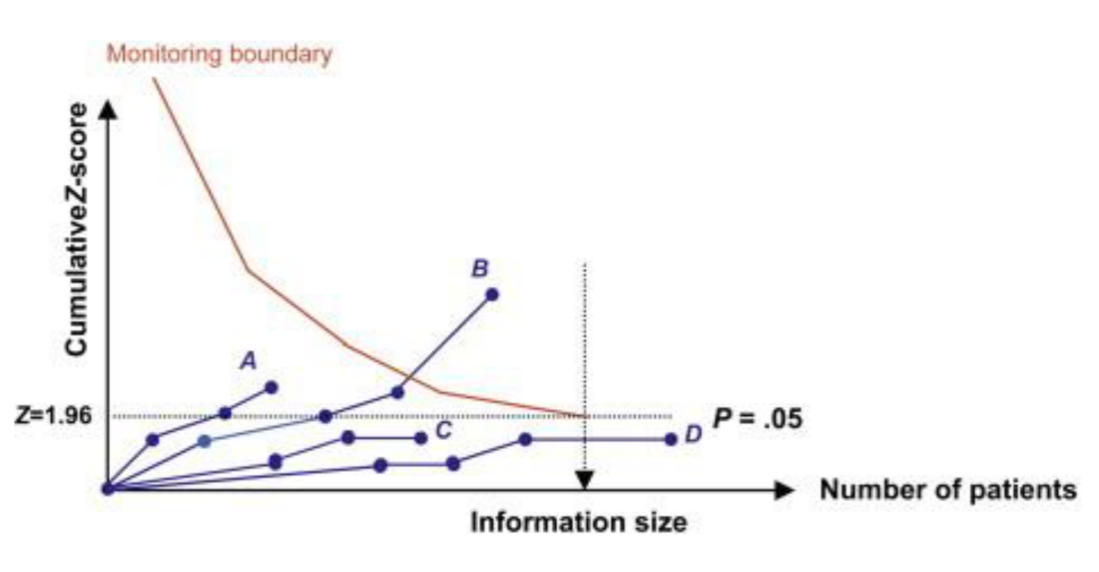
Trial sequential analysis.

We examined the reliability and conclusiveness of the available evidence using trial sequential analyses (TSA) [19-21]. The sample size required for a reliable and conclusive meta-analysis is at least as large as that of a single optimally powered RCT. Therefore, we calculated the sample size (ie, the heterogeneity-corrected optimal information size) required to detect or reject a minimal 25% relative risk reduction intervention effect. We then used the heterogeneity-corrected optimal information size to help construct Lan-DeMets sequential monitoring boundaries for our cumulative meta-analyses [22], analogous to interim monitoring in an RCT, to determine when sufficient evidence had been accrued (Figure 2): Significant (*P*<.05) meta-analysis included potentially spurious evidence of effect, that is, the cumulative Z-curve did not cross the monitoring boundaries (curve A), or firm evidence of effect, that is, the cumulative Z-curve crossed the monitoring boundaries (curve B). Nonsignificant (*P* ≥.05) meta-analysis included absence of evidence, that is, the meta-analysis included less patients than the required information size (curve C), or lack of effect, that is, the meta-analysis included more patients than the required information size (curve D). We conducted TSA using TSA version 0.937 with an intention to maintain an overall 5% risk of a type I error and 20% risk of a type II error (power of 80%).

## Results

### Study Selection and Characteristics of Included Studies

**Figure 3:**
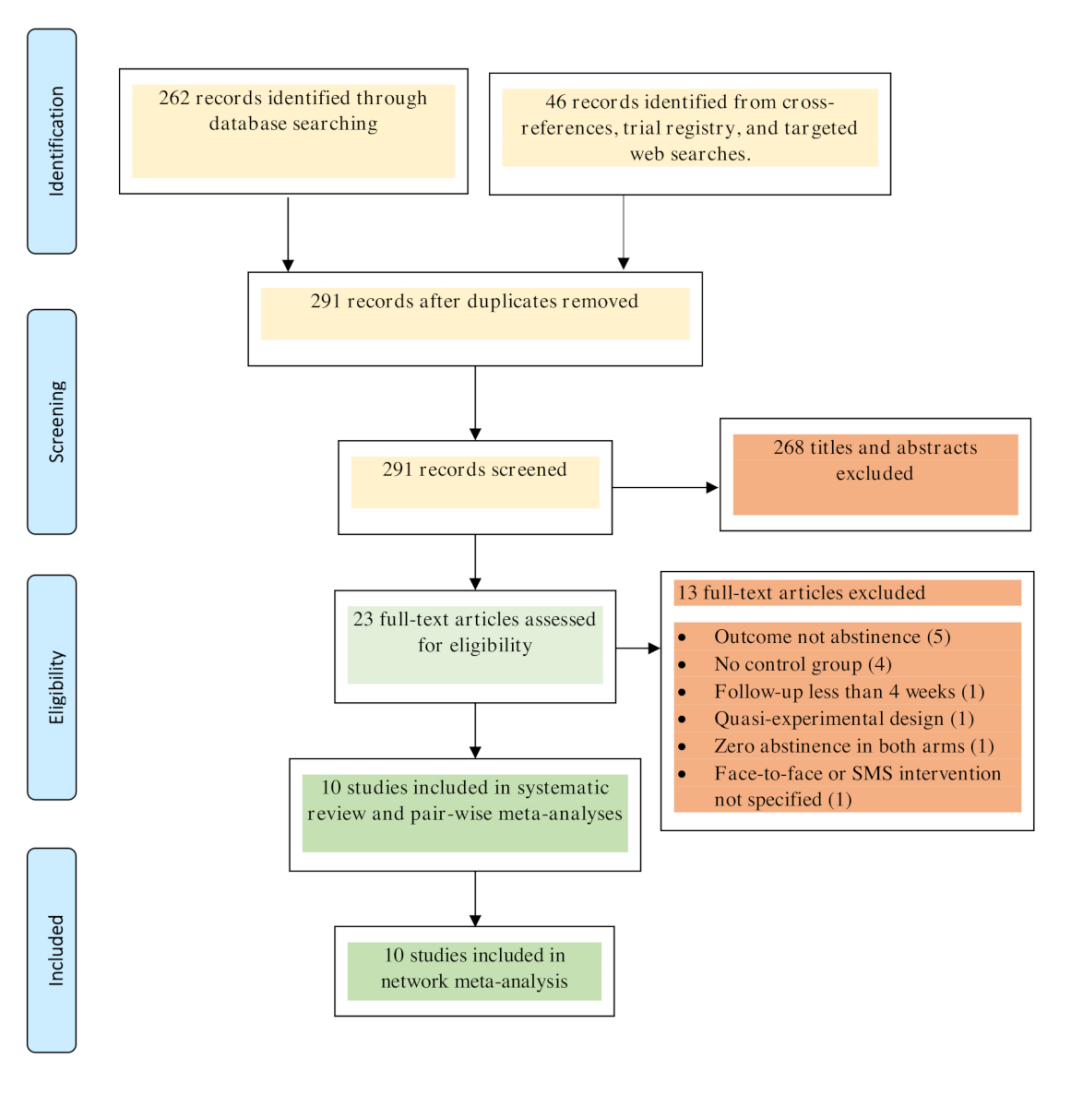
Study selection flow diagram. SMS: short message service.

The study selection process is illustrated in Figure 3. The search strategy yielded 308 records, and upon screening abstracts and duplicate records, we identified 23 potentially eligible studies. We excluded 13 additional studies for the following reasons: the outcome was not abstinence (4 studies), no control group existed (4 studies), follow-up was less than 4 weeks (1 study), the study was quasi-experimental (1 study), face-to-face or mHealth interventions were not specified (1 study), a zero smoking abstinence rate was found in both the intervention and control groups, a computational error was identified (1 study), and participants in the intervention arm received either mHealth or face-to-face interventions but not both (1 study). Hence, 10 RCTs met the inclusion criteria and were included in the analysis [23-32].

The 10 included studies comprised a total of 1772 participants, all of whom were current smokers living with HIV. All studies were from a high-income country, that is, the United States (Table 1). On average, each participant at baseline smoked about 16 cigarettes daily (range 11-20). The average age of the study population was 45 years (range 42-50), and women comprised about 37% (range 8%-100%). Smoking cessation strategies were administered face-to-face in 7 studies [23-27,29,30], and sustained smoking abstinence estimates were reported in 4 studies [23,30-32]. The intensity and maximum follow-up period ranged from 4 weeks to as much 52 weeks. The number of counseling also varied across the studies, from 1 session to 11 sessions.

### Risk of Bias in Included Studies

**Figure 4:**
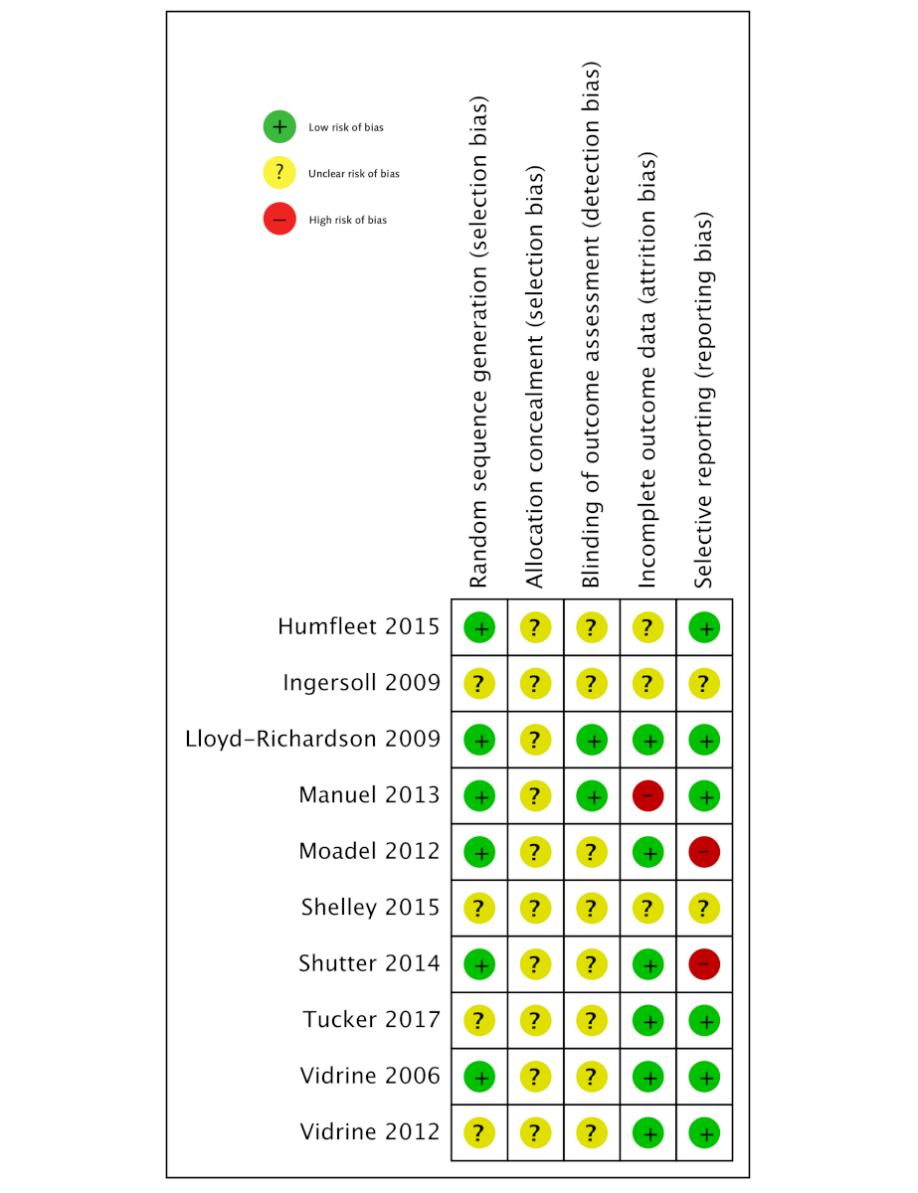
Risk of bias in included studies.

Among the 10 included studies, 6 reported the use of computer-generated lists of random numbers for randomization, whereas the other 4 studies did not describe the random sequence generation process. Allocation concealment was not described in any study; therefore, the risks of selection bias were unclear. Blinding of the participants and investigators was not described in 9 of the 10 studies, leaving 1 study in which investigators facilitated counseling sessions, which was thus judged to have a high risk of performance bias. Outcomes assessors were masked to the intervention in 2 studies, whereas the other 8 studies were assessed to have unclear risks of detection bias. Attrition bias was low in 7 studies, and reporting bias high or unclear in 5 studies (Figure 4).

### Short-Term Smoking Cessation (4 Weeks to <6 Months)

**Figure 5:**
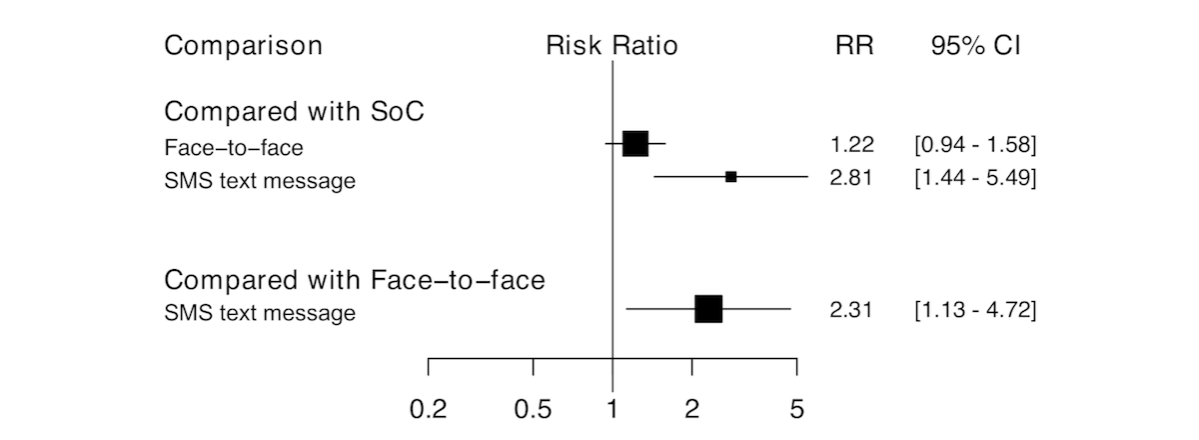
Pairwise comparisons of all interventions, short-term effect. RR: risk ratio; SMS: short message service; SoC: standard of care, no intervention control.

Figure 5 displays a caterpillar plot of the relative RRs and 95% CIs of efficacy for all possible pairwise comparisons of the different treatment strategies. For short-term smoking cessation (ie, ≥4 weeks of smoking abstinence within 6 months of the intervention), 7 trials compared face-to-face intervention versus no intervention control group, and 4 compared mHealth-delivered interventions versus no intervention control group (n=1870). Participants randomized to SMS-delivered interventions were 2.81 times more likely to have stopped smoking compared to those who received standard care (RR 2.81, 95% CI 1.44-5.49). In addition, PLHIV who received mHealth-delivered interventions were twice as likely to have stopped smoking compared to those who received face-to-face interventions (RR 2.31, 95% 1.13-4.72). On average, face-to-face interventions were no more effective than no intervention in increasing short-term smoking cessation (RR 1.22, 95% CI 0.94-1.58). The measure of inconsistency between studies (*I*^2^) was 6.3%, suggesting that the included studies were not statistically heterogeneous.

**Table 1:** Characteristics of the included studies.

Study	Sample size	Average age, years	Women (%)	Average daily number of cigarettes	Intervention	Outcome	Follow-up durations
Humfleet 2015 [23]	209	45	18	19.8	1 face-to-face CBT^b^ session and 6 face-to-face CBT sessions	Sustained smoking abstinence	12, 24, 26, and 52 weeks
Ingersoll 2009 [24]	40	42	45	17.3	1 face-to-face counseling session	7-day point prevalence smoking abstinence	4 and 12 weeks
Lloyd-Richardson 2009 [25]	444	42	37	18.2	4 face-to-face counseling sessions	7-day point prevalence smoking abstinence	2, 4, and 6 months
Manuel 2013 [26]	30	49	100	16.1	1 face-to-face counseling session	7-day point prevalence smoking abstinence	4 weeks
Moadel 2012 [27]	145	49	51	12.0	8 face-to-face counseling sessions	7-day point prevalence smoking abstinence	42 and 132 days
Shelley 2015 [28]	158	50	16	15.0	Twice daily short message service text messages and twice daily short message service text messages+7 phone counseling sessions	7-day point prevalence smoking abstinence	1, 4, 8, 12, and 24 weeks
Shuter 2014 [29]	138	46	40	10.9	8 face-to-face CBT sessions	7-day point prevalence smoking abstinence	6 weeks, 3 months
Tucker 2017 [30]	40	42.9	8	13.0	Face-to-face counseling	Sustained smoking abstinence	3 months
Vidrine 2006 [31]	94	43	22	20.1	8 phone counseling sessions	Sustained smoking abstinence	3 months
Vidrine 2012 [32]	474	45	30	19.2	11 phone counseling sessions	Sustained smoking abstinence	3, 6, and 12 months

^a^RCT: randomized controlled trial.

^b^CBT: cognitive behavioral therapy.

**Figure 6:**
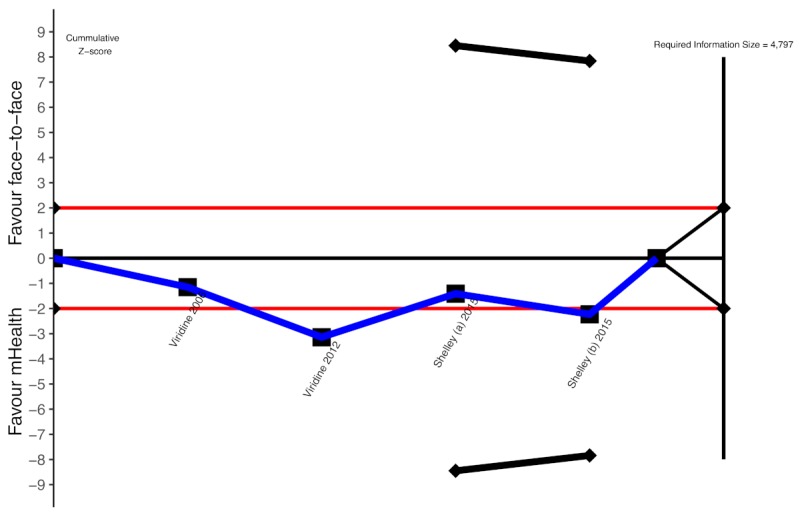
Trial sequential analysis for mHealth for smoking cessation in people living with HIV, short-term effect. mHealth: mobile health.

Our calculations indicated that the optimal information size needed to reliably detect a plausible treatment effect for short-term smoking cessation is 4797 participants (Figure 6). However, only 15.16% (726/4787) of the participants of the required information size were accrued. More so, the sequential monitoring boundary has not been crossed, indicating that the cumulative evidence is unreliable and inconclusive (Figure 6).

### Long-Term Smoking Cessation (≥6 Months)

**Figure 7:**
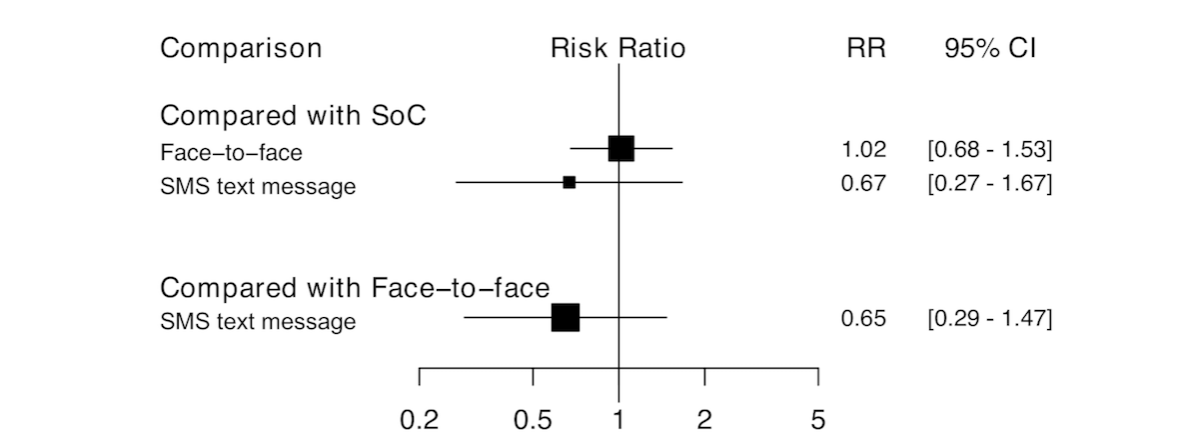
Pairwise comparisons of all interventions, long-term effect. RR: risk ratio; SMS: short message service; SoC: standard of care, no intervention control.

For long-term smoking cessation, that is, abstinence ≥6 months, 3 trials compared face-to-face intervention versus no intervention control group and 2 compared mHealth-delivered interventions versus no intervention control group (n=1546). There was no significant difference in smoking cessation rates between PLHIV randomized to mHealth-delivered interventions and those in the no intervention control group (RR 0.67, 95% CI 0.27-1.67). Similarly, there was no significant difference in smoking cessation rates between face-to-face interventions and no intervention control groups (RR 1.02, 95% CI 0.68-1.53). In addition, adjusted indirect treatment comparison between face-to-face and mHealth interventions revealed no significant difference (RR 0.65, 95% CI 0.29-1.47; Figure 7). The measure of inconsistency between the included studies (*I*^2^) was 0%, suggesting no evidence that the included studies were statistically heterogeneous.

**Figure 8:**
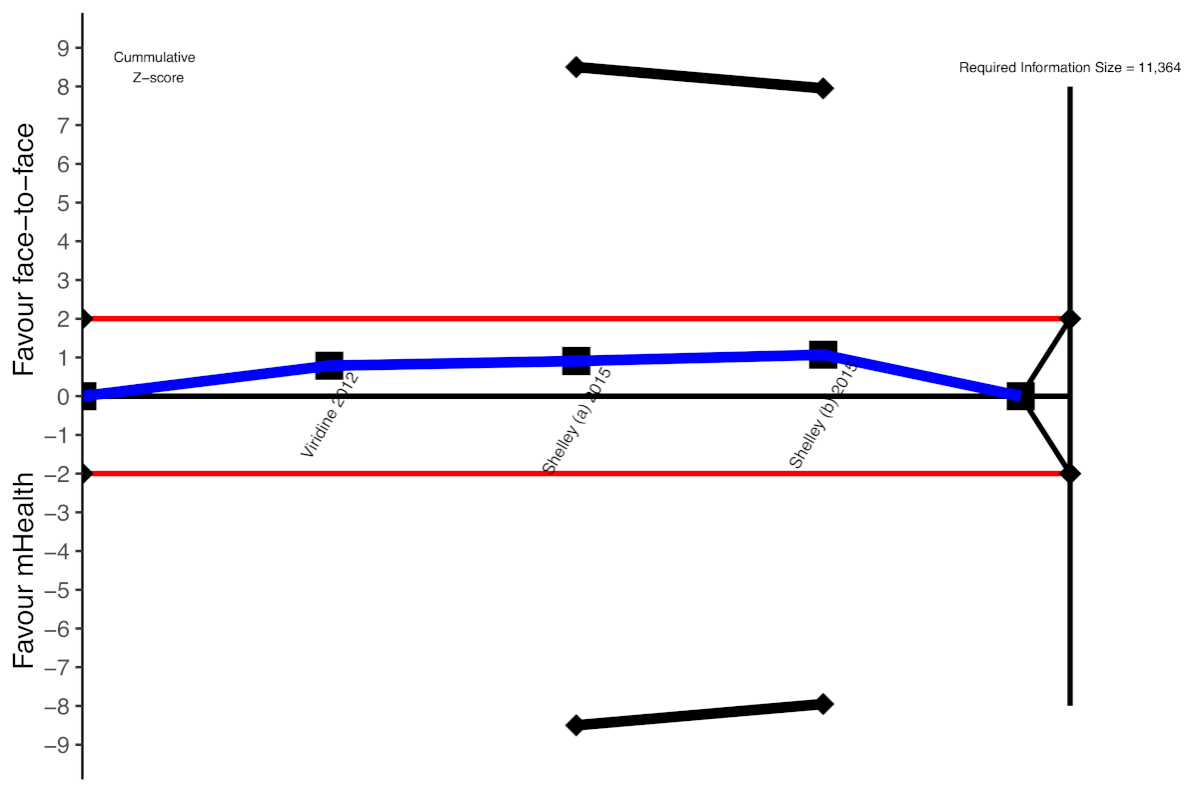
Trial sequential analysis for mHealth for smoking cessation in people living with HIV, long-term effect. mHealth: mobile health.

Our calculations indicated that the optimal information size needed to reliably detect a plausible treatment effect for long-term smoking cessation was 11,364 participants (Figure 8). However, only 5.56% (632/11,364) participants of the required information size were accrued in the pooled analysis. The sequential monitoring boundary has also not been crossed, indicating that the cumulative evidence is unreliable and inconclusive (Figure 8).

In Figure 8, dashed blue cumulative Z curves do not cross solid black trial sequential monitoring boundaries for benefit and horizontal red lines illustrate the traditional level of statistical significance (*P*=.05).

## Discussion

### Principal Findings

The results suggest that mHealth interventions for smoking cessation in PLHIV leads to better short-term improvement in smoking cessation rates than face-to-face interventions. However, from 6 months after the intervention and onward, there is no evidence of any effect regardless of the mode of intervention delivery. Our findings are broadly consistent with a previous meta-analysis of studies conducted in the general population, which reported a higher pooled smoking abstinence rate associated with SMS text messaging for 3 months compared to SMS text messaging for 6 months [33]. It is important to note that the absence of evidence is not evidence of absence of long-term effects of mHealth-delivered interventions. The lack of significant differences in long-term abstinence, however, may be due to the small number of studies contributing to this indirect evidence network, and as such, the evidence is inconclusive. Accurate understanding of the strength of the evidence for mHealth requires a systematic, comprehensive, and unbiased accumulation of the available evidence and methods adopted from formal interim monitoring boundaries applied to cumulative meta-analysis. The results of our TSA showed that the evidence that mHealth increases smoking cessation rates in the short term is encouraging but may be unreliable to make conclusive inferences.

Given the crucial need for the prevention of cardiovascular disease risk in PLHIV, there is a need for future pragmatic trials comparing mHealth and face-to-face intervention, especially in resource-limited settings that bear the highest burden of HIV and where smoking is now a bigger problem [2,34]. Furthermore, low-income settings are now experiencing an epidemiological transition from infectious diseases to chronic diseases [2] as a result of dramatic changes in diet and lifestyle. The epidemiological transition in resource-limited settings is happening over a shorter time frame than that experienced historically by high-income countries [34]. In addition, there is a need to identify mHealth-delivered interventions that are most beneficial for PLHIV. We should also investigate innovative specific features of mHealth interventions that can achieve long-term effects, for example, by varying the mode of delivery (weekly SMS text messaging) or by personalized and more tailored SMS text messages.

### Limitations

The limitations in our study warrant consideration. First, the included studies were conducted in a high-income setting, which potentially limits generalization of the results to low- and middle-income settings in which the burden of HIV and tobacco-related illnesses and deaths are currently most severe [5]. Nonetheless, our findings may be generalizable to other vulnerable groups in high-income countries. Second, the intervention arms in the included studies all comprised multicomponent strategies, which may have influenced our results; however, tests for heterogeneity revealed that the studies included in our analyses were in fact homogeneous. Third, we could not compare mHealth or face-to-face interventions with strategies that entailed a combination of both interventions because none of the included studies allowed this dual treatment in the intervention arm. Wewers et al [35] examined mHealth and face-to-face interventions in their study; however, we considered this study ineligible because the participants received either mHealth or face-to-face interventions and not both and because the numbers were not specified. Furthermore, with only 10 studies considered eligible for our review, we could not perform meta-regression analyses to explore potential effect-modifiers such as age, sex, coexisting substance abuse, and average number of cigarettes smoked daily at baseline. In spite of these limitations, we present novel systematic evidence evaluating the preferred mode of contact to be employed for improving smoking abstinence among PLHIV.

### Conclusion

Compared to face-to-face interventions, mHealth-delivered interventions can boost smoking cessation rates, at least in the short term, among PLHIV with higher smoking prevalence rates than the general population. However, it remains unclear how long the effects of such interventions last. Future research should focus on strategies for sustaining the treatment effect in the long term and move beyond high-income settings.

## Appendix

Multimedia Appendix 1 Medline search strategy.

## Abbreviations

mHealth: mobile health
PLHIV: people living with HIV
RCT: randomized controlled trial
RR: risk ratio
SMS: short message service
TSA: trial sequential analyses
