# Supplementary material for: Comparison of mHealth and Face-to-Face Interventions for Smoking Cessation Among People Living With HIV: Meta-Analysis
Source: JMIR Mhealth Uhealth. 2019 Jan 7;7(1):e203. doi: 10.2196/mhealth.9329 (PMC6329415; doi:10.2196/mhealth.9329)
Supplement: Multimedia Appendix 1 [file mhealth_v7i1e203_fig.ocx]

**Appendix 1. MEDLINE search strategy**

1. RANDOMIZED‐CONTROLLED‐TRIAL.pt.

2. CONTROLLED‐CLINICAL‐TRIAL.pt.

3. CLINICAL‐TRIAL.pt.

4. Meta analysis.pt.

5. exp Clinical Trial/

6. Random‐Allocation/

7. randomized‐controlled trials/

8. double‐blind‐method/

9. single‐blind‐method/

10. placebos/

11. Research‐Design/

12. ((clin$ adj5 trial$) or placebo$ or random$).ti,ab.

13. ((singl$ or doubl$ or trebl$ or tripl$) adj5 (blind$ or mask$)).ti,ab.

14. (volunteer$ or prospectiv$).ti,ab.

15. exp Follow‐Up‐Studies/

16. exp Retrospective‐Studies/

17. exp Prospective‐Studies/

18. exp Evaluation‐Studies/ or Program‐Evaluation.mp.

19. exp Cross‐Sectional‐Studies/

20. exp Behavior‐therapy/

21. exp Health‐Promotion/

22. exp Community‐Health‐Services/

23. exp Health‐Education/

24. exp Health‐Behavior/

25. or/1-24

26. smoking cessation.mp. or exp Smoking Cessation/

27. "Tobacco‐Use‐Cessation"/

28. "Tobacco‐Use‐Disorder"/

29. Tobacco‐Smokeless/

30. exp Tobacco‐Smoke‐Pollution/

31. exp Tobacco‐/

32. exp Nicotine‐/

33. ((quit$ or stop$ or ceas$ or giv$) adj5 smoking).ti,ab.

34. exp Smoking/pc, th [Prevention & Control, Therapy]

35. or/ 26-34

36. telephone.mp.

37. mobile.mp.

38. cellphone.mp.

39. "cell phone".mp.

40. sms.mp.

41. text*.mp.

42. or/ 36-41

43. exp hiv infections/ or exp acquired immunodeficiency syndrome/

44. hiv/ or hiv‐1/ or hiv‐2/

45. ("acquired immunodeficiency syndrome" or "acquired immunedeficiency syndrome" or "acquired immuno‐deficiency syndrome" or "acquired immune‐deficiency syndrome").mp.

46. "HIV/AIDS".mp.

47. HIV.mp.

48. PLWHA.mp

49. or/ 43-48

50. 25 and 35 and 42 and 49
